# Sex differences in features of atherosclerotic plaques as revealed by various imaging techniques: historical review

**DOI:** 10.3389/fphys.2025.1579885

**Published:** 2025-05-26

**Authors:** Dalya Laban, Alice Kattan, Lamia Ait-Abdellah, Hema Krishna, Elizabeth Le Master, Irena Levitan

**Affiliations:** ^1^ Division of Pulmonary and Critical Care, University of Illinois at Chicago, Chicago, IL, United States; ^2^ Division of Cardiology, University of Illinois at Chicago, Chicago, IL, United States; ^3^ Jesse Brown VA Medical Center, Chicago, IL, United States

**Keywords:** cardiovascular disease, vascular biology, plaque stability, fibrous cap thinning, sex differences

## Abstract

Numerous studies over several decades found that there are significant sex differences in the development and severity of atherosclerosis, which include plaque burden, composition and vulnerability to rupture. This review provides historical analysis of these studies starting with early histological analysis of *post mortem* samples to modern high-resolution imaging techniques. It is discussed that the abundance of evidence obtained by an array of approaches demonstrates that men are more prone to develop atherosclerosis, which manifests itself in earlier initiation of the plaques, while the occurrence of plaque is accelerated following menopause. These findings unequivocally show that men are more likely to develop plaques with larger lipid-rich necrotic cores, thinner fibrous caps, and stronger inflammatory responses, resulting in increased vulnerability at a younger age. However, the rapid escalation of plaque instability in postmenopausal women, which is caused by a significant reduction in smooth muscle cell density and changes in calcification patterns, results in comparable atherosclerotic burden in men and women in older adults. These findings highlight how differences in sex and age, influence the development and severity of atherosclerosis. Understanding these differences is essential for creating better ways to assess and treat heart disease in men and women.

## Introduction

Atherosclerosis is a chronic inflammatory disease of the arterial walls caused by the buildup of plaque made up primarily of cholesterol-laden foam cells, as well as cholesterol and calcium depositions, and cellular debris. Depending on the severity grade of the plaques, they can narrow the arteries and block the blood flow, causing serious cardiovascular events, such as stroke and heart failure. Notably, atherosclerosis shows significant differences between men and women including the size of the plaques, their composition and clinical outcomes (Pasterkamp et al., 2022; Poznyak et al., 2023; Van Dam-Nolen et al., 2023). In general, plaque prevalence is higher in men than in women at younger ages, but this difference mostly disappears after women reach menopause (Burke et al., 2001; Seegers et al., 2022). There are also significant differences between men and women in the plaque composition that affect plaque stability, which is a key factor in the cardiovascular risk, with more vulnerable plaques found in younger men than in women of the same age but this difference mostly disappears in older adults. The clinical symptoms of cardiovascular disease are also more heterogenous in women (Seegers et al., 2022). An increase in plaque vulnerability in older women and the heterogeneity of clinical presentation may lead to a delayed diagnosis and treatment, and consequently, a more severe outcome. This review provides an overview of the studies that addressed the differences in plaque burden and vulnerability over the last several decades using an array of approaches, which developed over time.

## Plaque prevalence and progression

**FIGURE 1:**
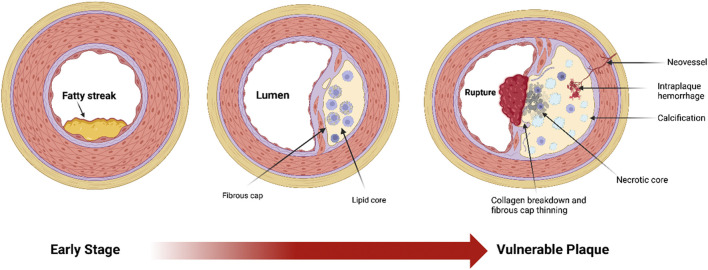
Diagram of three major stages of atherosclerotic plaque development, starting with early stage fatty streak formation, to its most vulnerable stage, including thinning of the fibrous cap and collagen breakdown, a large necrotic core, foam cells (lipid-laden macrophages), spotty calcifications, intraplaque hemorrhage, and plaque neovascularization.

Plaque burden refers to the total amount of atherosclerotic plaque accumulated on arterial walls, an important factor for cardiovascular risk assessment and treatment strategies. Plaque buildup develops in stages, beginning with the formation of fatty streaks, the earliest visible signs of lipid accumulation in the arteries, which progress over time to fibrous plaques—thicker, more complex lesions that can constrict blood flow (Figure 1). A major factor in determining clinical outcomes is plaque vulnerability or its sensitivity to rupture, as it is the plaque rupture and subsequent acute thrombotic events that lead to most severe outcomes, including heart attack and stroke (Carr et al., 1996; Costopoulos et al., 2017; Lovett et al., 2004; Spagnoli et al., 2004). The feature that determines plaque stability is the relationship between the fibrous cap and the soft plaque core: a thick cap composed of collagen and smooth muscle cells create a barrier over the lipid core, protecting it from rupture (Harman and Jørgensen, 2019; Holzapfel et al., 2002; Lopes et al., 2013). Stable plaques are characterized by thick fibrous caps with abundant smooth muscle cells, contributing to a dense, collagen-rich structure (Davies, 1996; Doran et al., 2008; Lee and Libby, 1997). Furthermore, smooth muscle cells secrete an extracellular matrix that further stabilizes the plaques (Doran et al., 2008; Lee and Libby, 1997). In contrast, plaques rich in soft fat and lower density of smooth muscle cells and thin caps tend to rupture, especially in areas where the fibrous cap is the thinnest (Davies, 1996; Tracy, 1997). Multiple studies demonstrated significant differences between men and women in plaque prevalence, progression and vulnerability in an age-dependent way.

**FIGURE 2:**
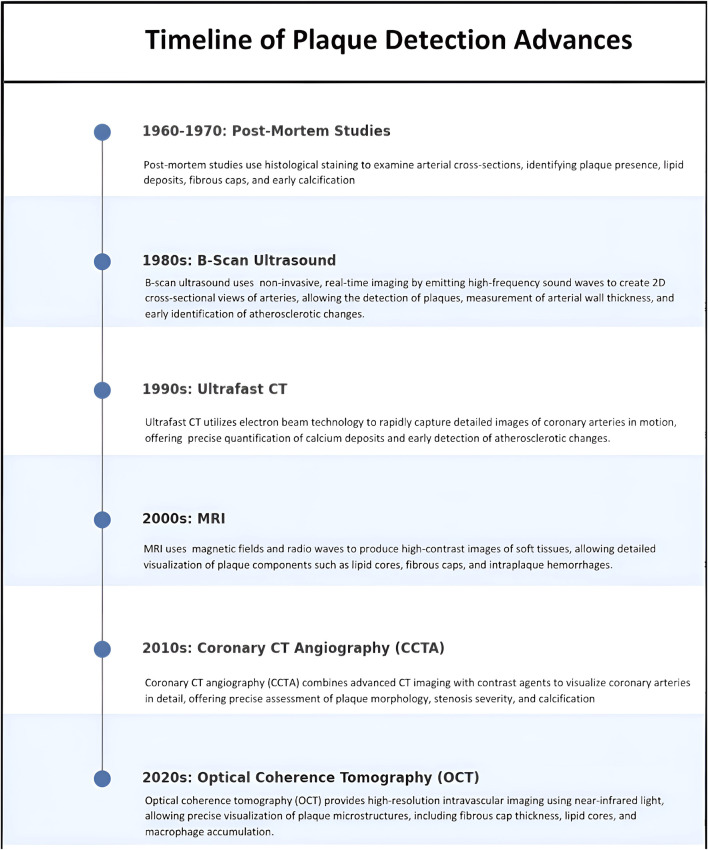
Timeline of advancements in plaque detection techniques from the 1960s to the 2020s. The progression highlights the evolution of imaging modalities, starting with histological post-mortem studies and advancing to modern techniques such as optical coherence tomography (OCT).

Higher plaque burden and faster progression in men compared to women have been reported by numerous studies using different approaches over several decades as described in Figure 2. Very early studies identified increased plaque burden in men compared to women in a relatively small cohort of patients (∼100 men and women), which was associated with the differences in the heart mass, which was first proposed to affect the development of atherosclerosis (Roberts and Roberts, 1980; Roberts and Buja, 1972). However, multiple later studies found that the differences in the plaque burden between men and women cannot be explained by the differences in the heart sizes. It has also been known for decades that fatty streaks form at a young age, as early as at ages 10–14 teenagers (Vanĕcek, 1976; Velican and Velican, 1979). Interestingly, Vanĕcek, 1976 found that fatty streaks appeared earlier in girls compared to boys, with 41% of girls and 24% of boys developing fatty streaks in coronary arteries when they are teenagers but a progression of fatty streaks to fibrous plaque was faster in men than in women: fibrous plaque appeared in most men by the age of 40 and in most women by the age of 50 (Vanĕcek, 1976). A similar conclusion was reached in a large autopsy study over 23,000 individuals across various race and location groups: progression from early to more advanced lesions happened faster in males compared to females, with the rate of prevalence increasing with age, regardless of geographic or racial differences (Tracy and Toca, 1977).

Development of non-invasive imaging techniques, such as ultrasound, allowed evaluating plaque burden in patients during the progression of the disease. Overall, the conclusions of these studies regarding the sex differences in plaque progression were consistent with the earlier reports: men have higher plaque burden and develop plaques at younger age. For example, in MONICA project that examined over 1,000 patients, men and women aged 25 to 65, it was revealed that men tend to develop plaques earlier and also have higher prevalence than women (Gostomzyk et al., 1988). Similarly, a study of a cohort of middle-aged men and women with known coronary artery disease (CAD), found that men showed more plaque formation in coronary arteries than women and were more likely to have plaques developing earlier in life, typically in their early to mid 40s (Li et al., 1994).

**FIGURE 3:**
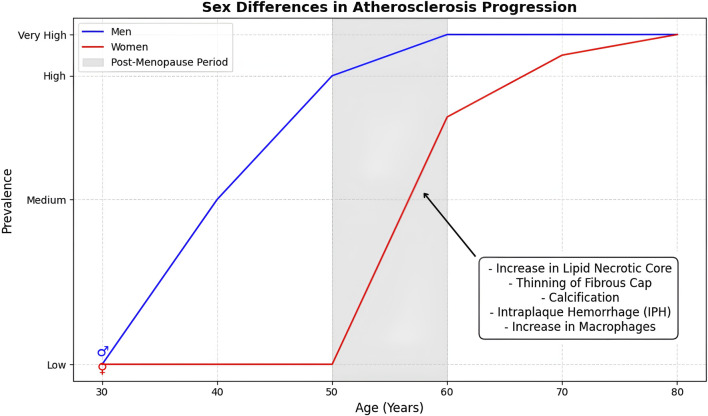
Age-dependent sex differences in atherosclerosis progression. The graph illustrates that men develop atherosclerosis earlier with a higher prevalence at younger ages, while the prevalence in women accelerates post-menopause, leading to convergence in older age.

Further population-based research has shown that the difference between men and women in plaque prevalence narrows and disappears with age. In a large cohort of both sexes (>3,000 men and >3,000 women), ranging from 25 to 84 years old, it was found that 55% of men and 46% of women had atherosclerotic plaques in carotid arteries across all ages, but the predominance of atherosclerosis in men declined after the age of 50, when the prevalence in women increased faster than in men (Joakimsen et al., 1999). More specifically, atherosclerotic burden increased linearly in men until the age of 65, after which it plateaued, whereas in women, the progression was slower than in men until the age of 49, after which it accelerated. In elderly men and women (75–84 years old) the prevalence of carotid plaques was found to be similar and even slightly higher in women, even though the difference was not statistically significant. A reason for the studies’ variance in plaque prevalence of older men and women could have been survivor selection-bias favoring atherosclerosis-free males (Joakimsen et al., 1999). Thus, older men with lower rates of plaque burden were more likely to be overrepresented. Consistent with this study, more recently, Ruiz-Garc et al. (2012) found a significant sex difference in plaque burden in men vs. women younger than 65 years old with men developing a higher number of plaques with larger volumes, but no difference in patients of 65 and older. In both men and women, there was a significant increase in plaque burden with age with a narrowing difference between the sexes (Figure 3). Notably, a recent study based on UK Biobank study, showed that the cardioprotection is significantly diminished in women by smoking. Furthermore, it was found that women who smoke are disproportionately at a higher risk for developing peripheral arterial disease (PAD) compared with males (Xu et al., 2023). Female smokers in the United Kingdom, for instance, had an 18% higher risk for PAD.

Mechanistically, it was shown that sex hormones play major role in the protective effect against plaque progression in women. For example, Hodis et al. (2001) found that the progression of atherosclerosis was significantly lower in the group of postmenopausal women receiving 17*β* estrogen as compared to the placebo group. Interestingly, 17*β* estrogen therapy did not have significant impact in women who were taking lipid lowering medications suggesting that the effect of estrogen may be mediated via normalization of the lipoprotein profiles. Consistent with this idea, Xie et al. (2022) suggested that estrogen mediates its atheroprotective effects through estrogen receptor α (Erα) and its regulation of a key transcription factor in lipid metabolism SREBP-1. As a crucial regulator of lipid metabolism, nuclear SREBP-1 controls the expression of lipid-related target genes, while cytoplasmic retention of SREBP-1 suppresses cholesterol and lipid synthesis pathways, reducing intracellular lipid accumulation in the liver. Under normal conditions, SREBP-1 nuclear expression remains low; however, hypercholesterolemia significantly increases its nuclear localization. Xie et al. (2022) found that treating hypercholesterolemic ovariectomized mice with 17*β* estrogen promotes the cytoplasmic retention of SREBP-1 providing strong evidence for this pathway to be involved. They also found that there is a significant correlation between the expressions of estrogen receptor and SREBP-1 in blood and livers of human genomic database. Other possible mechanisms include protective effects for endothelial and vasodilatory functions via phosphorylation signaling cascades, such as Mitogen-activated protein kinases (MAPK) signaling (Razandi et al., 2000). Estrogen also enhances cardiomyocyte function by promoting vasodilation, increasing angiogenesis, improving cell survival, and reducing reactive oxidative species (Iorga et al., 2017). Moreover, estrogen-replacement therapy in postmenopausal women is associated with improved heart failure survival, and reduction of infarct size and amount of cardiomyocyte apoptosis in animal models of myocardial infarction (Patten et al., 2004).

Interestingly, several studies found that not only estrogen but also the male sex hormone, testosterone, has a cardioprotective effect. This conclusion came from the studies showing that: (i) low testosterone levels were found to be associated with higher degrees of CAD (Rosano et al., 2007); (ii) men exhibit increased risk of the coronary arterial disease as testosterone begins to decline around age 40 (Oskui et al., 2013; Goodale et al., 2017); (iii) postmenapuasal women with reduced levels of plasma testosterone have the highest risk for major adverse cardiovascular events (MACE) compared to women with normal levels, suggesting that testosterone provides a cardioprotective effect also in postmenopausal women (Islam et al., 2022). Apparently though, the cardioprotective effects of testosterone are significantly lower than those of estrogen, which would explain the observed sex differences in cardioprotection and atherosclerosis development.

## Plaque composition and vulnerability

Most acute cardiovascular events including heart attacks and sudden deaths result from plaque ruptures. Multiple studies found that the composition of the plaques, which determine the likelihood of rupture is significantly different between men and women. The stability of the plaques is determined by the fibrous cap, which covers the necrotic core of the plaque and consists of smooth muscle cells (SMC) embedded into the extracellular matrix, in particular collagen: the thickness and the density of the cap determine the probability of rupture (Alonso-Herranz et al., 2023). The thinning or degradation of the cap, which leads to increased vulnerability to rupture, may occur as a result of the senescence and loss of SMCs and/or the breakdown/decrease in the deposition of collagen (Alonso-Herranz et al., 2023). Mechanically, vulnerable plaques with larger necrotic core and thinner caps are soft, whereas stable plaques with thicker caps are stiff, a feature used to assess plaque vulnerability. Also, a major biomarker of plaque instability is intraplaque hemorrhage (IPH), which results from a rupture of neovessels in plaque formed as the plaque progresses and known as a major complication of plaque development (Levy and Moreno, 2006). These key factors, along with sex-based differences in smooth muscle cell loss, fibrous cap thickness, and calcficiation, are summarized in Table 1.

**TABLE 1:** Sex-based determinants of plaque vulnerability in atherosclerosis.

Plaque vulnerability factor	Role in plaque vulnerability	Sex differences	Reference
Smooth muscle cell loss (SMCs)	Found in extracellular matrix of plaque caps and an increase in SMCs contributes to cap stability	Men experience a more rapid loss of SMCs, resulting in a greater breakdown of the structural integrity of plaques	Tracy (1997)
Fibrous plaque thickness	Thinner fibrous caps are more prone to rupture compared to thick caps	Men often experience thinning of fibrous caps, while women experience thinner caps later than men	Alonso-Herranz et al. (2023)
			Burke et al. (2001)
			Tracy (2001)
Echolucency/lipid content	Higher echolucency indicates lipid-rich areas which are associated with plaque instability	Echolucent plaques are more common in men, but plaque size differences between males and females are not statistically different	Mathiesen, et al. (2001)
Inflammatory response	Increased presence of inflammatory cells such as macrophages can lead to an increase in plaque vulnerability	Women exhibit greater accumulation of macrophages compared to men	Kinoshita et al. (2024)
Intraplaque hemorrhage (IPH) presence	Blood from ruptured neovessels leaks into plaque core, causing an increase in plaque volume and an immune response, leading to increased vulnerability	IPH sizes do not differ between men and women, but IPHs are more prevalent in men	Van Dam-Nolen et al. (2023)
Necrotic core size	The presence of, and increases in, necrotic core size heighten the risk of plaque rupture and instability	Necrotic core sizes do not differ between men and women, but necrotic cores are more prevalent in men. Difference in prevalence narrowed and disappeared post menopause	Van Dam-Nolen et al. (2023)
Calcification	Accumulation of calcium deposits in the plaques and arterial walls leads to increase in plaque vulnerability. A calcium score can serve as a indicator of heart health	Females under 65 exhibited less calcification than men. Men exhibit higher calcium scores than women on average	Oliver et al. (1964)
			Janowitz et al. (1993)
			Plank et al. (2019)
			Lessmann et al. (2019)
			Nakao et al. (2018)
			Shaw et al. (2018)

Summary of key changes associated with atherosclerosis progression in men, including increased lipid necrotic core, thinning of the fibrous cap, calcification, intraplaque hemorrhage (IPH), and macrophage accumulation.

An early study by Tracy (1997) provided histological analysis of atherosclerotic plaques in coronary arteries of men and women 15–54 years old obtained from autopsies. They showed a decrease in SMCs content of the plaques in both men and women but with a significant difference in the rate of SMC loss: men showed faster rate of the loss of SMC, and consequently reached a point of plaque instability at a younger age. More specifically, men reached the threshold for atherosclerosis on average in the early 40s, while women reached the same point in the mid-50s, with more than a 10-year delay. This delay in plaque vulnerability in women could be attributed to their ability to maintain a higher density of smooth muscle cells for a longer time (Tracy, 1997). An increased propensity of plaques to rupture in men was also reported in patients who underwent carotid endarterectomy to treat carotid stenosis, with a mean age of the patients 71 years old (Carr et al., 1997). In this study, men constituted 86% in a group with ruptured plaques and 59% in a group with no rupture, which was associated with the thinning of the fibrous cap and more severe inflammatory response, as evidenced by an increase in the presence of macrophages and T-lymphocytes, all assessed by histological analysis of the tissues removed during the surgeries. There are also sex differences in vascular wall remodeling of coronary arteries with age (Tracy, 2001). The study examined the medial thickening of coronary arteries, which is known to correlate with atheroma formation. Analyzing the autopsies of 370 subjects (both male and female) aged 15–79 years, they found that as coronary arteries age, both the intima and the media thicken, which might be attributed to the buildup in collagen depositions, with men having a faster progression of media thickening than women in the age groups of 54 years old and younger, which indicates that men have higher predisposition to atherosclerosis in this age group. In the older group, however, of 55–79 years olds, this trend seems to be reversed with the proportion of women developing significant thickening of the media increasing, but the number of subjects in this age group is relatively small.

Further insights into plaque vulnerability are provided by ultrasound imaging that measures plaque echolucency measuring sound propagation. This approach discriminates between *echolucent* (darker) plaques, which are softer and tend to have higher fat content from *echogenic* (bright) plaques that are stiffer and are associated with more fibrous tissue and calcification (European Carotid Plaque Study Group, 1995; Nordestgaard et al., 2003).Using this approach, Joakimsen et al. (1999) found that the prevalence of soft plaques increases with age in both men and women in parallel, until it reaches a plateau at ∼ 60 years of age, and that men have higher prevalence of soft plaques than women in all age groups, even though the difference does not appear to be statistically significant in most age groups. In another study (Tromsø Study), Mathiesen et al. looked at differences in plaque composition in the arteries according to sex and possible relation to risk for future cardiovascular events. It was observed that similarly to the previous reports men had more echolucent plaques, which are more prone to rupture, than women (7% and 3.2%, respectively in the population with the average age of 67–68, ∼800 people), even though the difference was not statistically significant (Mathiesen et al., 2001). In a larger and more recent study by Li et al. (2021), which focused on high-risk individuals for stroke, 2,644 participants aged 40 years or older were examined using similar ultrasound techniques to assess carotid plaque vulnerability. Consistent with the previous reports, Li et al. (2021) observed that vulnerable plaques were significantly more common in men (20.0% in men vs. 12.8% in women).

A similar conclusion was reached in the study by Kinoshita et al. (2024) who examined a sex-specific link between perivascular inflammation and plaque vulnerability. Perivascular inflammation was evaluated through the attenuation of pericoronary adipose tissue observed via computed tomography angiography, while plaque vulnerability was determined using optical coherence tomography (OCT). A multivariable regression approach was employed to examine how levels of perivascular inflammation and plaque burden influenced the identification of OCT features indicative of vulnerability. This analysis adjusted for other factors, including age, clinical presentation, hypertension, hyperlipidemia, diabetes, chronic kidney disease, and smoking status. The findings showed that women exhibited a higher prevalence of thin-cap fibroatheroma and greater macrophage accumulation related to perivascular inflammation, indicating higher plaque vulnerability. Moreover, in women, perivascular inflammation correlated with the presence of thin-cap fibroatheroma and increased macrophage accumulation, while no such association was found in men, suggesting a role for perivascular inflammation in distinct pathobiological processes based on sex (Kinoshita et al., 2024).

In a more detailed analysis of plaque features, Van Dam-Nolen et al., 2022 used magnetic resonance imaging (MRI) and multidetector computed tomographic angiography (MDCTA) on 156 men and 68 women (69 mean age) with ischemic cerebrovascular symptoms and carotid stenosis to study any sex-specific differences in plaque structures and composition. MRI has the capability to identify different markers found on plaques such as necrotic cores, soft plaques, hard plaques, IPH, severe stenosis, and ulcerations (Tartari et al., 2011). After adjustment of plaque volume differences, there were no sex specific differences in IPH size, lipid rich necrotic core size, or calcification size, but 73% of men had lipid rich necrotic cores, while only 41% of women displayed lipid rich necrotic cores. Similarly, men exhibited IPH more often than women. Men also experienced a “trifecta” of coexisting characteristics more often than women: men more often exhibited calcifications, lipid rich necrotic cores and IPH all together or thin/ruptured fibrous caps, lipid rich necrotic cores and IPH all together (Van Dam-Nolen et al., 2022).

It is also well established that progression of the plaques and increase in plaque vulnerability with age are accelerated in women, suggesting that it might lead to steep increase in heart problems in aged women. Specifically, Burke et al. (2001) analyzed plaques of 51 women who died suddenly from coronary artery disease, and discovered that postmenopausal women (over 50) had significantly more vulnerable plaques than premenopausal women. These vulnerable plaques in older women were characterized by thinner fibrous caps and larger lipid cores, both of which make the plaques more likely to rupture. Furthermore, a cross-sectional study of 906 patient MRIs from 2005 to 2014 found that while men were more likely to develop carotid IPH than women, as women aged post-menopause, the development of IPH converged with that of men (Singh et al., 2017). Consistent with these studies, Seegers et al. (2022) found that women exhibited a marked acceleration in the formation of lipid-rich plaques with age, rising from 26% in women under 50 years old to 83% in those over 80 in a large cohort of men and women (1,368 patients) with acute coronary syndrome (ACS). Similarly, the prevalence of thin-cap fibroatheroma increased significantly in women, from 16% in those under 50% to 43% in women over 80. This age-related progression was not observed in men. Additionally, women showed a higher prevalence of cholesterol crystals, macrophages, and calcification, which increased by up to 20% with advancing age, also not observed in men.

Notably, while it is well established that women develop more stable atherosclerotic plaques than men, the molecular mechanisms responsible for these effects are still poorly understood. One possibility is the differential production interferons, cytokines that may have multiple effects on the cardiovascular system. For example, it was found that a relative resistance of females as compared to males to malaria could be attributed to interferon-gamma (IFN-y) (Cernetich et al., 2006), a cytokine that was shown earlier to inhibit proliferation of vascular smooth muscle (Amento et al., 1991). Women were also found to produce higher levels of type I interferon, as compared to males (Webb et al., 2018) but the effects of type I interferon on the cardiovascular system are complex and depend on multiple factors (Tran et al., 2024). New insights into the genetic basis of the sex differences in atherosclerosis development were recently provided using single-cell RNA sequencing of the atherosclerotic arteries of men and women, which identified cell-specific differences in gene expression, particularly in the smooth muscle cells (Hartman et al., 2021). These studies provide strong basis for further mechanistic analysis.

## Calcification and its impact on plaque stability

Plaque vulnerability in atherosclerosis, or the likelihood of plaque rupture, is strongly influenced by calcification, the buildup of calcium in the artery walls. This process often starts with tiny calcium deposits, called microcalcifications, within the intima (Akers et al., 2019; Mori et al., 2018) and gradually merge into larger clusters as the disease progresses, forming calcified fragments that affect the plaque’s stability (Huang et al., 2001). While larger calcium deposits can sometimes help stabilize the plaque, smaller, spotty deposits within thin, fragile areas of the plaque can increase stress, raising the risk of rupture (Otsuka et al., 2014). Therefore, while calcification is generally a marker of advanced disease, its impact on plaque stability depends on where it forms and how it spreads within the artery wall (Wong et al., 2012). Calcifications on the outer edges of the necrotic core may provide some structural support, while smaller, fragmented deposits within thinner regions of the plaque can increase vulnerability to rupture, as these smaller fragments are known as microcalcifications (Shioi and Ikari, 2018). Over the decades, studies examined the distribution of calcification with different techniques in order to determine how calcification could lead to rupture. Using fluoroscopy, Oliver et al. (1964) demonstrated that calcium deposits increased with age in both sexes in individuals with and without ischemic heart disease (IHD): among the subjects who did not have IHD, females below 65 years showed less calcification compared to their male counterparts but among the IHD group there was no difference in calcification between the sexes. Using ultra-fast CT scans, which offered much higher detail and a qualitative measure of coronary calcium compared with fluoroscopy, Janowitz et al. (1993) found that, overall, women tended to have lower amounts of coronary calcium compared with men, particularly before age 60 years. The difference between the sexes tended to narrow after 60 years of age, further suggesting that age is a factor in decreasing the difference in calcium between sexes. No significant differences between the ages of 40–69 years were found for men in calcium scores, and from 50 to 79 years for women (Janowitz et al., 1993). Consistent with these early studies, more recently, studies from the Million Veteran Program (2023) have shown that postmenopausal women with low estrogen levels have higher levels of coronary artery calcium (CAC) development, which is a measurement of the areas of calcium deposition and density in the coronary arteries. Using non-invasive imaging scans, a CAC score is obtained to determine calcium build up and associated risk of heart disease, with 1–10 being low risk and 101–300 score being severe calcium build up and high risk of adverse cardiovascular events. Therefore low estrogen levels in postmenopausal women are associated with a higher CAC score indicating an increased risk of adverse cardiovascular outcomes (Nguyen et al., 2024). As women’s plaque characteristics begin to match those of men, the initial sex disparities in CAC may become less noticeable by the age of 60.

These early studies, considered seminal in retrospect, provided the foundation for the development of the CAC score in the early 1990s as our understanding of plaque calcification evolved. Although initial calcium measurements in the past depended on basic X-rays and fluoroscopy, today clinicians are mostly using ultrafast CT scans that better quantify the build-up. Using coronary computed tomography angiography in 1,050 patients (525 males, 525 females), Plank et al. (2019) showed that there were significantly higher CAC scores in males, with a mean of 180.5 Agatston Units (AU), compared to 67.8 AU for females (Plank et al., 2019). Similar studies further confirm that males have a higher score of calcification than females, and as a predictor of a heart problem (Lessmann et al., 2019; Nakao et al., 2018; Shaw et al., 2018).

However, while men have greater CAC scores than women, the Framingham Heart Study of 2011 found that the women were at greater cardiovascular risk than the men who had the same level of scores, suggesting calcification in women can reflect more severe vascular aging and not merely serve as a marker for stable plaque (Fernández-Friera et al., 2015). This is important because CAC has traditionally been employed to signify plaque stability, particularly in men, but could have a different implication in women. Furthermore, the Van Dam-Nolen et al. (2022) study provides strong evidence supporting the distinction between calcification patterns in men vs. women with men having greater calcification volumes while women having more diffuse, widespread calcification.

Most recently, there is increasing interest to employ Artificial Intelligence, (AI)-driven analysis, to enable faster and more accurate interpretation of imaging scans, to reduce human error and strengthen risk assessment. AI analysis is based on machine learning (ML) and deep learning (DL) algorithms to improve the detection capabilities. Several studies recently demonstrated high degree of success, ranging between >70% and >90% in detecting atherocelrotic plaques (Zreik et al., 2019; Rajendra Acharya et al., 2019; Xu et al., 2022). Furthermore, beyond plaque detection, AI algorithims can also assess plaque vulnerability and inflammatory status (Oikonomou et al., 2018; Oikonomou et al., 2019). These new developments are expected to provide highly improved strategies for diagnosis and treatment.

## Clinical implications

Though the lessened quantity and vulnerability of plaque does confer an overall lower risk profile in pre-menopausal women, clinicians should remain alert to anginal symptoms in this population. Women less often complain of typical chest pain and present on average an hour later to the emergency room following symptom onset, leading to delays in time to treatment. Compared to male peers, young women experience a near two-fold increase in early acute coronary syndrome mortality (Mehilli and Presbitero, 2020).

Driving these outcome disparities, women presenting with acute coronary syndrome are more likely to experience non plaque-rupture coronary events, such as spontaneous coronary artery dissection, vasospastic angina, or myocardial infarction with non-obstructive coronary arteries (MINOCA). Mechanisms of MINOCA and its counterpart, ischemia with non-obstructive coronary arteries (INOCA), are yet poorly understood, but encompass coronary thrombosis and embolism, dyfunction of the coronary microvasculature, and coronary spasm (Pasupathy et al., 2015). Over one in three women diagnosed with MINOCA will demonstrate plaque rupture or ulceration under IVUS (intravascular ultrasound) imaging, suggesting some traditional atherosclerotic mechanisms are at play (Reynolds et al., 2011; Burke et al., 2003). Diagnosis can be made via intracoronary imaging and physiology testing with IVUS, OCT, fractional flow reserve, coronary flow reserve, index of microvascular resistance, and vasoreactivity testing. Non-invasive techniques including stress positron emission tomography and stress cardiac magnetic resonance imaging with myocardial blood flow reserve may also be used (Gulati et al., 2021). Women with MINOCA (Patel et al., 2006) and INOCA exhibit increased risk of death, MI, rehospitalization, and need for repeat angiography compared to those with normal coronaries (Jespersen et al., 2012; Maddox et al., 2014; Gulati et al., 2009). A high clinical index of suspicion is required, not only because additional diagnostic techniques are required, but also because of important differences in treatment. For example, spontaneous coronary artery dissection is preferentially treated without percutaneous coronary intervention, due to risk of propogating the dissection plane, and specific classes of medications, such as calcium channel blockers, are of particular value in vasospastic angina (Saw et al., 2019; Hassan et al., 2019; Gowda et al., 2024).

Not only are women predisposed to differing mechanisms of coronary artery disease, they also experience unique risk modifiers in developing traditional atherosclerosis. Often overlooked in clinical practice, a history of pre-eclampsia in pregnancy, onset of early menopause, polycystic ovary, fibromuscular dysplasia, and higher co-incident rates of autoimmune and inflammatory conditions can all accelerate plaque formation, resulting in adverse cardiovascular events at younger ages.

Improvements in diagnostic techniques, treatment, and importantly in awareness of heart disease among women lead to a substantial decline in associated mortality between 1980 and 2010, though more recently, women under age 65 have seen a rise in heart disease deaths (Khan et al., 2022). Continued efforts to understand sex differences in vascular health are vital to reversing this trend.

## Conclusion

Predisposition to atherosclerosis is significantly influenced by sex and age: the preponderance of evidence shows that men tend to develop higher plaque burden with greater vulnerability at younger ages than women, but this difference diminishes or disappears with age. The first evidence of these differences came from early histological studies demonstrating higher lipid depositions and stenosis in younger men vs. women, which is associated with the development of cardiovascular disease. As more sophisticated imaging techniques developed, similar conclusions were reached based assessing the density or softness of plaque material using ultrasound and on viewing calcifications using CT scans. Further technological advancements provided further insights into the differences in plaque features between men and women by identifying plaque markers and composition with MRI, 3D imaging of coronary arteries using coronary computed tomography angiography, and assessing macrophages and thin cap fibroatheroma for indications of plaque vulnerability using OCT. However, it is also abundantly clear that as women reach menopause, the progression of atherosclerosis accelerates based on the increased development of lipid-rich plaques, thin cap fibroatheroma, calcifications, cholesterol crystals, and macrophages. Thus, oftentimes, women displaying significant plaque vulnerability later in life results in delayed diagnosis and treatment.

To prevent delays in care for postmenopausal women, it is important to develop tailored approaches that can identify and address atherosclerosis progression earlier and more effectively. While technological advancements have improved the imaging and quantification of plaque development, further advancements could enhance specificity. New technologies could identify high-risk plaque features and disease pathways based on diagnosis and provide accurate quantification of fibrous cap thickness, microcalcifications, and lipid core size.

Notably, though development into the molecular pathways of pro-atherodevelopment have emerged, especially in relation to sex-hormones and inflammation markers, still, more studies are required to develop a more clear molecular pathway of which can be a target for therapeutics. One way to facilitate this research is to both develop and utitlize the current genomic studies which have a wealth of signaling data. Moreover, the development of additional animal models of atherosclerosis, including lipid and inflammation targets, would target and focus the current body of data regarding the role of sex-differences in atherosclerosis progression and adverse cardiovascular events. Furthermore, our breadth of knowledge of sex differences in atherosclerotic development would benefit from additional studies focusing on race and sex/race interactions.
